# By-Laws of the Dental Protective Association of the United States

**Published:** 1889-01

**Authors:** 


### By-Laws of the Dental Protective Association of the United States.

OFFICERS: HOW ELECTED.

SECTION I. The Officers of the Association shall consist of a President, Vice-President, Treasurer, Secretary, and Board of Directors, who shall hold their respective offices for one year, and until their successors are elected and qualified: PROVIDED, that any officer may be removed by the Board of Directors whenever the interests of the Association shall require, and the Board of Directors shall be the sole judges of the interests of the Association: AND PROVIDED FURTHER, that the office of Vice-President and Secretary may, at the discretion of the Board, be held by one and the same person.

ANNUAL ELECTION.

SEC. II. The annual meeting of members shall be held in Chicago, on the Third Monday in December of each year. A notice designating the place of such meeting shall be signed by the Secretary and published in a public newspaper in Chicago, and in such of the Dental Journals as the Board of Directors shall select, and the Secretary shall also mail a written or printed notice to each member at least ten days before said day.

SEC. III. A Board of Directors, who must be members of the Association, shall be elected annually at the annual meeting of members, by the members to serve for one year, and until their successors shall have been elected.

SEC. IV. The Board of Directors shall consist of three members of the Association, to be selected and chosen by a majority vote of all the members of the Association. Every member shall have the right to vote at the regular Annual Meetings for three Directors, either personally or by proxy, and in case of the member not appearing personally at the regular Annual Meeting to vote and no one being designated as proxy for him, the President and in his absence, the Vice-President shall be authorized to vote as proxy for such member.

MEETING OF DIRECTORS.

SEC. V. Immediately after the annual election of Directors, the Board of Directors elected, shall meet and select a President, Vice-President, Secretary and Treasurer, of whom the President, Vice-President and Secretary shall be members of the Board of Directors.

### SPECIAL MEETINGS OF THE ASSOCIATION.

Sec. VI. The President and Board of Directors may call special meetings of the members of the Association, which shall be held at its general office at such time as shall be designated in the call; and, in such case, it shall be the duty of the Secretary of the Association, at least ten days before the time fixed for holding such meeting, to mail to each member entitled to vote, whose address is known to the Secretary, a notice specifying the time and place of holding said meeting, and briefly stating the object of the meeting, if the same has been mentioned in the call; and the Secretary shall also cause to be published in a public newspaper in Chicago, and in such of the Dental Journals as the Board of Directors may name, at least ten days prior to the time of holding the meeting, a notice of like effect.

### REGULAR MEETINGS OF THE BOARD OF DIRECTORS.

Sec. VII. The Board of Directors of this Association shall hold their regular meeting at its general office in Chicago on the fourth Monday of each month at the hour of 4 o'clock, P. M. A majority of the Board shall constitute a quorum for the transaction of all business; and in case there be no quorum present on the day fixed for their regular meeting, the member or members present may adjourn the meeting from time to time until a quorum be obtained, or may adjourn said meeting *sine die.*

### SPECIAL MEETINGS OF THE BOARD OF DIRECTORS.

Sec. VIII. The President of the Board of Directors shall have the power to call special meetings of the Board, whenever he deems it expedient so to do; and it shall be his duty to call special meetings of the Board at the request of one Director of the Association; in either instance a notice of time and place of meeting shall be served or mailed to each Director, giving at least one day's notice thereof, exclusive of the day on which said notice is mailed.

DUTIES OF PRESIDENT AND VICE-PRESIDENT.

SEC. IX. The President, and in his absence the Vice-President, shall preside over all meetings of the Association and over all meetings of the Board of Directors at which he may be present, to preserve order and regulate discussion according to parliamentary usage.

The Vice-President shall, in the absence of the President, perform all the duties of the President. (In case of absence of the President and Vice-President from any meeting of the Association, a President *pro tem* may be chosen to preside).

DUTIES OF SECRETARY.

SEC. X. The Secretary shall attend all meetings of the Association and Board of Directors, whether regular or special; he shall keep, in a book prepared for that purpose, a true record of the proceedings of all such meetings. He shall have the custody of the corporate seal, and shall attach the same to all documents which require sealing.

DUTIES OF TREASURER.

SEC. XI. The Treasurer shall receive, have charge of and disburse, upon proper vouchers the funds of the Association under the direction of the Board of Directors, and he may be removed at any time, by the Board of Directors, for any misconduct in the affairs of his office, and may be required to give bonds for such amount as the Board of Directors may designate, with one or more sureties to be approved by the Board of Directors.

DIRECTORS, AND THEIR POWERS.

SEC. XII. The Board of Directors shall consist of three members, to whom the policy, conduct, property and affairs of the Association and its membership is hereby confided. The Board of Directors shall have power:

To accept or reject applications for membership in the Association.

To fill any vacancy that may occur in any office.

To levy and collect, if necessary, assessments which, in all, shall not exceed ten dollars.

To take entire charge of the defense of members of the Association in any of the States or Territories, when prosecuted for the infringement of patents, the validity of which has not been fully established, and with the funds of the Association to retain and pay one or more counsel of their own selection, and with the funds of the Association to pay and defray all necessary and proper expenses of any such litigation.

### MEMBERSHIPS.

SEC. XIII. Subject to the approval of the Board of Directors, any member of the Dental Profession may become a member of the Association on payment to the Treasurer of a membership fee of ten dollars, and subscribing to the By-Laws of the Association.

### VACANCIES.

SEC. XIV. Whenever a vacancy shall occur in the Board of Directors, from any cause, it shall be filled for the remainder of the term by the Board of Directors.

### BOARD OF DIRECTORS TO MAKE REPORT.

SEC. XV. At the regular annual meeting of the members for the election of Directors, the retiring Board shall make, through their officers, a full report of the condition of the Association, including a financial statement showing all assets and liabilities, and the amount of money received and disbursed during the year.

SEC. XVI. It is understood that all officers of the Association are amenable to the Board of Directors, supreme authority upon all points affecting the Association or its management being vested in said Board.

### ALTERATION OR AMENDMENT OF BY-LAWS.

SEC. XVII. These By-Laws may be altered or amended at any regular meeting of the Board of Directors, by a vote of a majority of all the Directors: PROVIDED, that a copy of such proposed alteration or amendment shall have been submitted to the Board in writing, at least one week previous to the adoption thereof.

Sec. XVIII. Upon all questions of construction of By-Laws the decision of the Board of Directors shall control until overruled by a vote of the Association. Any By-Law may be suspended by two-thirds vote of any regular meeting of the Association.

Sec. XIX. The presence of ten members shall be necessary to constitute a quorum at any meeting of the Association; which number may be increased or diminished by any subsequent By-Law.

Sec. XX. At each stated meeting of the Association the order of business shall be as follows:—

1. Reading of minutes of preceeding meeting.
2. Reports of officers.
3. Reports of Committees.
4. Unfinished Business.
5. New Business.

Any one desiring to become a member of the Association can do so by notifying any one of the officers.

